# Mr. Taunton on Hernia

**Published:** 1812-07

**Authors:** John Taunton

**Affiliations:** Surgeon to the City of London Truss Society, the City and Finsbury Dispensaries, and Lecturer on Anatomy and Surgery. 21, Grenville Street, Hatton Garden


*To the Editors of the Medical and Physical Journal.*

GENTLEMEN,

THE following statement of the situation and occurrence of Hernia, at different periods of life, has been obtained principally from patients relieved by the City of London Truss Society,* within the short period of four years and a half, and entirely under my own observation; it appeared to me to form an interesting article of reference to the medical, philosophical, and general, reader; as such, I have taken the liberty of transmitting it for publication in your valuable Journal, if it meets your approbation.

* See the following account of the Charity.

In

In 3176 patients, 2702 were males, and 474 were females. The situation of the Hernia, in each case, will be seen in the following Table:

| | | | |
|---|---|---|---|
| 704 | Left Inguinal | 1910 Inguinal | 2194 Single |
| 1206 | Right Inguinal | | |
| 154 | Left Femoral | 284 Femoral | |
| 130 | Right Femoral | | |
| 728 | Double Inguinal | .......... | 792 Double. |
| 64 | Double Femoral | | |
| 172 | Umbilical | ........... | 190 |
| 18 | Ventral | | |
| 3176 | | | 3176 |

| | | | |
|---|---|---|---|
| 210 | Patients were relieved with Trusses | under | 10 years of age. |
| 160 | ditto | . . . . . . . between | 10 and 20 ditto. |
| 310 | ditto | . . . . . . . . . . . . . | 20 and 30 ditto. |
| 596 | ditto | . . . . . . . . . . . . . | 30 and 40 ditto. |
| 632 | ditto | . . . . . . . . . . . . . | 40 and 50 ditto. |
| 664 | ditto | . . . . . . . . . . . . . | 50 and 60 ditto. |
| 432 | ditto | . . . . . . . . . . . . . | 60 and 70 ditto. |
| 168 | ditto | . . . . . . . . . . . . . | 70 and 80 ditto. |
| 10 | ditto | . . . . . . . . . . . . . | 80 and 90 ditto. |
| 2 | ditto | . . . . . . . . . . . . . | 90 and 100 ditto. |
| 3176 | | | |

From the most accurate estimation which I have been enabled to make, I have no doubt of this malady existing in one person in eight through the whole male population of this kingdom; and even in a much greater proportion among the laboring classes of the community, in manufacturing districts, particularly in those persons who are employed in weaving.

JOHN TAUNTON,
Surgeon to the City of London Truss Society, the City and Finsbury Dispensaries, and Lecturer on Anatomy and Surgery.

21, *Grenville Street, Hatton Garden, June* 1812.

*City of London Truss Society, for the Relief of the Ruptured Poor throughout the Kingdom, Grocers' Hall Court, Poultry. Instituted* 1807.

The objects of this Charity are to provide trusses for every kind of rupture—to furnish bandages and other necessary instruments for all cases of prolapsus—to perform every necessary operation—to administer surgical aid promptly—and to supply medicines and attendance during the cure of the patient.

It is a well known and justly-lamented fact, that a very considerable portion of our fellow-creatures are suffering, in various degrees, under the disease called Hernia, or Rupture.

This malady occurs at all periods of life, in either sex, and is not the consequence of depraved habits, but arises either from bodily defect, or those laborious exertions, from which the affluent are, in general, exempted. Hence a very large portion of the sufferers under this affliction is found among the poorer classes of the community.

When cases of Hernia are neglected in their early stages, either through delicacy, or extreme poverty, the malady daily increases in proportion to the industrious habits of the patient; and, from this circumstance, the services of many ingenious artificers and useful laborers have been totally lost to the community; and their families, once decently maintained by them, are now absolutely impoverished!!

There is, therefore, no disease which more imperiously calls for the charitable aid of a liberal public, and to the honor of human nature; such a call has not been made in vain! Since the institution of Societies, specifically for the relief of this malady, (the remedy for which, when judiciously applied, is peculiarly simple, and infallibly certain,) many afflicted persons have been encouraged by the prospect of *gratuitous* advice and assistance, to make known a calamity, which they had before unwisely concealed—have obtained the requisite relief—their lives have been spared—and their usefulness to the public continued.

